# Identification of hub genes related to CD4^+^ memory T cell infiltration with gene co-expression network predicts prognosis and immunotherapy effect in colon adenocarcinoma

**DOI:** 10.3389/fgene.2022.915282

**Published:** 2022-08-29

**Authors:** Lingxue Tang, Sheng Yu, Qianqian Zhang, Yinlian Cai, Wen Li, Senbang Yao, Huaidong Cheng

**Affiliations:** ^1^ Department of Oncology, the Second Affiliated Hospital of Anhui Medical University, Hefei, China; ^2^ Department of Oncology, Anhui Medical University, Hefei, China

**Keywords:** colon adenocarcinoma, immunotherapy, CD4 + memory T cell, weighted gene coexpression network analysis, gene

## Abstract

**Background:** CD4^+^ memory T cells (CD4^+^ MTCs), as an important part of the microenvironment affecting tumorigenesis and progression, have rarely been systematically analyzed. Our purpose was to comprehensively analyze the effect of CD4^+^ MTC infiltration on the prognosis of colon adenocarcinoma (COAD).

**Methods:** Based on RNA-Seq data, weighted gene co-expression network analysis (WGCNA) was used to screen the CD4^+^ MTC infiltration genes most associated with colon cancer and then identify hub genes and construct a prognostic model using the least absolute shrinkage and selection operator algorithm (LASSO). Finally, survival analysis, immune efficacy analysis, and drug sensitivity analysis were performed to evaluate the role of the prognostic model in COAD.

**Results:** We identified 929 differentially expressed genes (DEGs) associated with CD4^+^ MTCs and constructed a prognosis model based on five hub genes (F2RL2, TGFB2, DTNA, S1PR5, and MPP2) to predict overall survival (OS) in COAD. Kaplan–Meier analysis showed poor prognosis in the high-risk group, and the analysis of the hub gene showed that overexpression of TGFB2, DTNA, S1PR5, or MPP2 was associated with poor prognosis. Clinical prediction nomograms combining CD4^+^ MTC-related DEGs and clinical features were constructed to accurately predict OS and had high clinical application value. Immune efficacy and drug sensitivity analysis provide new insights for individualized treatment.

**Conclusion:** We constructed a prognostic risk model to predict OS in COAD and analyzed the effects of risk score on immunotherapy efficacy or drug sensitivity. These studies have important clinical significance for individualized targeted therapy and prognosis.

## Introduction

Colon cancer is one of the most common gastrointestinal malignancies in humans. GLOBOCAN 2020 estimated 1,148,515 new cases and 5,76,858 deaths due to colon cancer, ranking fifth among 36 cancers globally, accounting for 6 and 5.8%, respectively (Sung et al., 2021). Among all histological subtypes, colorectal adenocarcinoma accounts for more than 90% of colon cancer types (Barresi et al., 2015). Surgery and chemotherapy have improved the overall survival (OS) of colon cancer to a certain extent, but postoperative recurrence and emergence of acquired drug resistance have affected the prognosis of patients. The development of colon cancer is a multistep process caused by gradual accumulation of mutations in tumor suppressor genes, oncogenes, and epigenetic changes (Migheli and Migliore, 2012). In recent years, with in-depth exploration of tumor markers and biomarkers, clinical treatment decisions for colon cancer have changed greatly. A series of markers have been shown to play an important role in the early diagnosis of cancer, monitoring the efficacy of treatment and follow-up of possible recurrence. It is now easier to choose the most appropriate strategy for managing colon cancer (Lech et al., 2016). For example, microsatellite instability-high (MSI-H) was shown to be a predictor of improved overall survival (OS), and chromosome 18q deletion was associated with worse prognosis (Popat and Houlston, 2005; Kim et al., 2007). Patients with mutations in the tumor suppressor gene p53 had better OS when treated with adjuvant chemotherapy than those treated with surgery alone (Russo et al., 2005). KRAS mutation was confirmed to be correlated with non-responsiveness to cetuximab and panitumumab (Di Fiore et al., 2007), and BRAF mutations make patients resistant to anti-EGFR monoclonal antibodies and predict worse prognosis (Roth et al., 2010). Although targeted therapy has been incorporated into the treatment regimen for colon cancer, there is currently no comprehensive drug selection strategy to identify patients who will benefit the most. Therefore, it is of great significance to construct diagnostic and predictive biomarker models to identify the best prognostic biomarkers and help the selection of therapeutic drugs.

The occurrence and development of cancer are closely related to the complex tumor microenvironment (TME). Immune cells in the immune system which contain immune parameters related to survival are an important component of the microenvironment (Galon et al., 2013). Recently, several studies have confirmed that the molecular profile of immune-related genes in TMB may be a promising biomarker for predicting OS in cancer patients. (Huang R et al., 2020). In general, understanding the interaction of cancer and immune cells can help patients assess whether they would benefit from clinical treatment, especially immunotherapy. At present, the choice of treatment options and prognosis evaluation of colon cancer mainly depend on pathological tissue type, TNM stage, and biomarkers (Angell et al., 2020). Although these prediction methods are widely used in clinical practice, they still cannot provide complete prognostic information. For example, patients with the same histological tumor stage may have a significantly different clinical prognosis. Therefore, individualized treatment can maximize the benefits and minimize the harm to patients, resulting in optimal survival status and relatively long survival time.

T cell immunity is a hot research topic in recent years. CD4^+^ memory T cells (CD4^+^ MTCs) are closely associated with the prognosis in breast cancer (Deng et al., 2019), gastric cancer (Ning et al., 2020), lung adenocarcinoma (Choi and Na, 2018), and pancreatic cancer (Gu et al., 2020), but the role in colon adenocarcinoma (COAD) is unclear. Common detection methods for immune infiltration include flow cytometry and immunohistochemistry, but they cannot comprehensively measure the immune effects of different immune cell types. The wide application of high-throughput sequencing makes transcriptomic data more accessible and provides large amounts of resources for the analysis of immune cell infiltration (Lv et al., 2022). Predictive biomarker screening based on a database has been widely used in various diseases and achieved good results, especially in cancer-related fields. Weighted gene co-expression network analysis (WGCNA) is a comprehensive biological analysis method used to describe the correlation pattern between genes in microarray samples and pairwise relationships between gene transcripts. WGCNA can also be used to find clusters or modules of highly related genes, analyze correlations between modules and clinical characteristics, and identify biomarkers or therapeutic targets (Langfelder and Horvath, 2008; Yang et al., 2021a). This method has been successfully applied to analyze gene expression data from various types of cancers, such as breast cancer (Tian et al., 2020), lung cancer (Ding et al., 2019), melanoma (Wan et al., 2018), hepatocellular carcinoma (Yang et al., 2021a), glioblastoma (Zhou J et al., 2021), oral squamous cell carcinoma (Yang et al., 2021b), and ovarian cancers (Su et al., 2021). Compared with other signature construction methods, WGCNA pays more attention to the strong associations between genes and can more accurately identify the prognostic-related hub genes, which provides a novel method for us to construct a higher-resolution prognostic model and a new idea for predicting disease prognosis (Panahi and Hejazi, 2021). As a feature selection method, the least absolute shrinkage and selection operator (LASSO) is increasingly used in colon. We usually use LASSO regression analysis to mitigate the over-fitting of genes with prognostic value (Narala et al., 2021). To improve the accuracy of prediction and the generalization of statistical models, LASSO eliminates unnecessary covariates in a combined nonlinear and interactive manner (Obermeyer and Emanuel, 2016; Pavlou et al., 2016). Compared to traditional statistical models, LASSO has a better ability to identify key predictors of clinical features.

In this study, we screened the CD4^+^ MTC-related differentially expressed genes (DEGs) by WGCNA and then used LASSO-Cox regression analysis to identify hub genes and construct a prognostic model. We used the prognostic model to predict OS and established nomograms to improve prediction capacity. We also discussed the specific role of CD4^+^ MTC-related hub genes in colon adenocarcinoma (COAD) and predicted the effectiveness of immunotherapy and potential therapeutic drugs. Finally, we explored the function and biological signaling pathway of CD4^+^ MTC-related genes. These results will provide more precise treatment strategies for prognosis of colon cancer.

## Materials and methods

### Data collection and preprocessing

437 transcriptome data files (including 398 COAD and 39 normal samples), 385 clinical data files, and 399 gene mutation data files of the colon cancer training set were downloaded from the Cancer Genome Atlas database (TCGA, https://portal.gdc.cancer.gov/). The retrieval strategy is shown in Supplementary Table S1. Public microarray data and clinical data of the testing set (GSE40967-GPL570) were downloaded from the Gene Expression Omnibus database (GEO, https://www.ncbi.nlm.nih.gov/geo/) by using the keywords “colon cancer,” “survival,” and “*Homo sapiens*”. Then, we use Perl software for preliminary processing of the TCGA data. We first extracted gene expression data from transcriptome files of 398 tumor samples. These data were then analyzed to identify DEGs between normal and COAD samples. Second, the clinical features of each sample were extracted from 385 clinical samples, including survival status, OS, age, gender, and TNM stage. Subsequently, we merged the gene expression data and clinical data based on the sample IDs and finally obtained a total of 379 training set samples (Supplementary Table S2 **)**. Similarly, we screened 579 samples with complete clinical features from 585 GEO samples and merged them with the microarray data (Supplementary Table S3 **)**. In addition, we also obtained tumor mutational burden (TMB) data from 399 samples (Supplementary Table S4 **)**. TMB is defined as the total number of somatic gene coding errors, base substitutions, gene insertions, or deletion errors detected per million bases (Yarchoan et al., 2017). Finally, the cytotoxic T-lymphocyte-associated protein 4 (CTLA-4) and programmed cell death protein 1 (PD-1) immunotherapy score data were downloaded from the Cancer Immunome Database for further analysis (TCIA**,** https://tcia.at/).

### Immune cell infiltration and co-expression network construction

The “CIBERSORT” (Newman et al., 2015) algorithm was used to quantify the tumor-infiltrating immune cells from cancer RNA-seq data, and the relative proportions of 22 types of immune infiltrating cells were determined from the reference data set (the gene expression characteristic set of 22 immune cell subtypes) (Chen et al., 2018). Also, we searched the genes in normal samples and COAD circularly to identify the DEGs between the two groups. Both |logFC| ≧ 1.0 and adjusted *p* < 0.05 were used as the thresholds for DEGs. Based on the results of immune cell infiltration in DEGs, a co- expression network was constructed by R package “WGCNA”(18) and Pearson correlations (Botía et al., 2017) to understand correlation patterns between genes and identify important modules associated with COAD. In order to meet the requirements of scale-free topology, the soft threshold method was used to evaluate the correlation coefficient and noise filtering capability. The optimal soft threshold power β is determined by the function in WGCNA. The topological overlap matrix (TOM) and corresponding dissimilarity matrix (1-TOM) visualize the network graph for module detection. Subsequently, a scale-free topology plot was generated under the optimal soft threshold power, and a clustering tree of co-expressed gene modules was established, with the main parameters as cutHeight = 10,000, minSize = 10. DEGs closely related to 22 types of immune infiltrating cells were obtained by WGCNA to represent the expression profiles of module genes.

### Identification of hub genes and construction of a prognostic model

We extracted the target module of CD4^+^ MTC infiltration DEGs from the co-expression network according to the *p*-value of the correlation coefficient. The correlation was deemed significant when the false discovery rate (FDR) was *p* < 0.05 (Chen et al., 2021). Based on the gene expression in TCGA and GEO preprocessing results, the expression levels of target module DEGs in each sample were obtained. Then, we performed a univariate Cox regression analysis on the TCGA training set to screen out DEGs which were significantly associated with OS (Supplementary Table S5). Subsequently, LASSO regression analysis was performed on these DEGs to remove genes that were highly correlated and prevent the overfitting of the model. Next, DEGs with the least error in LASSO regression were determined by cross-validation. Based on the results of LASSO, we constructed a Cox model to obtain the prognostic hub genes and model formula. Then, the risk score was calculated using the model formula, and the training set was divided into high- and low-risk groups according to the median value of the risk score. Similarly, the GEO validation set was also divided into high- and low-risk groups to verify the accuracy of the prognostic model.

### Predictive ability of the model

We validated the prognostic value of the model by Kaplan–Meier (K–M) analysis. The accuracy of the prognostic model in predicting 1-, 3-, and 5-year OS rates was assessed by time-dependent receiver operating characteristic (ROC) curve. In addition, multivariate and univariate Cox regression analyses were performed to verify whether the risk score could be used as a prognostic indicator independent of other clinical features. We also performed survival analysis for each hub gene to determine the impact of their differential expression on the prognosis of COAD. Furthermore, based on the results of independent prognostic factors, we used “regplot” and “rms” packages to draw nomograms and calibration curves. Each prognostic factor corresponds to a score, and the scores of all prognostic factors were added to obtain a total score. Then, the total score was used to predict the 1-, 3-, and 5-year survival of COAD, and the calibration curve was used to verify the accuracy of the nomogram.

### Correlation analysis of risk score with immune cell infiltration, immunotherapy, and drug sensitivity

Based on pan-cancer immune cell infiltration data from the TCGA database (Supplementary Table S6), the correlation of immune cells with risk scores was analyzed. We also analyzed the correlation of each hub gene with immune cell infiltration and immune checkpoint. After downloading the CTLA-4 and PD-1 immunotherapy scores of COAD patients downloaded from the TCIA website, we compared the difference in immunotherapy efficacy between high- and low-risk groups. The higher the immunity score, the more patients benefit from immunotherapy. Finally, the pre-prepared installation package “pRRophetic” was used to predict drug sensitivity for COAD. The “pRRophetic” package is mainly used to predict the phenotype and drug sensitivity of external cell lines by using gene expression data. It can also be used to predict clinical data. (i.e., predicting clinical outcomes based on the cancer genome project cell coefficient). The half-maximal inhibitory concentration (IC_50_) value is used to represent the sensitivity of drugs. The smaller the IC_50_ value, the more sensitive the patient is to the drug.

### Functional and pathway enrichment analysis

We used the R package “limma” package and logFC function to analyze the genes in the prognostic model and screened out DEGs between high- and low-risk groups. LogFC >0 means the gene is upregulated in the high-risk group, and conversely, it is upregulated in the low-risk group. Subsequently, Gene Ontology (GO) and Kyoto Encyclopedia of Genes and Genomes (KEGG) (Dennis et al., 2003) enrichment analyses were performed based on the data obtained from risk analysis. The FDR (< 0.05) correction was used to determine the statistical significance of GO and KEGG terms.

### Tumor mutational burden analysis

We analyzed the sorted gene mutation data with the R package “maftools” and generated the corresponding waterfall diagram according to the high- and low-risk groups, showing the 30 genes with the highest mutation frequency. R software was used to verify whether there was a difference in TMB between high- and low-risk groups, and the association between TMB and OS was verified by K–M analysis.

### Statistical analysis

R (v 4.1.3) (https://www.r-project.org/) and SPSS 23 were used for statistical analysis, and Strawberryperl (v 5.30.0) (https://www.perl.org/) was used to sort and merge the downloaded data.

## Results

### Evaluation of tumor-infiltrating immune cells

**FIGURE 1:**
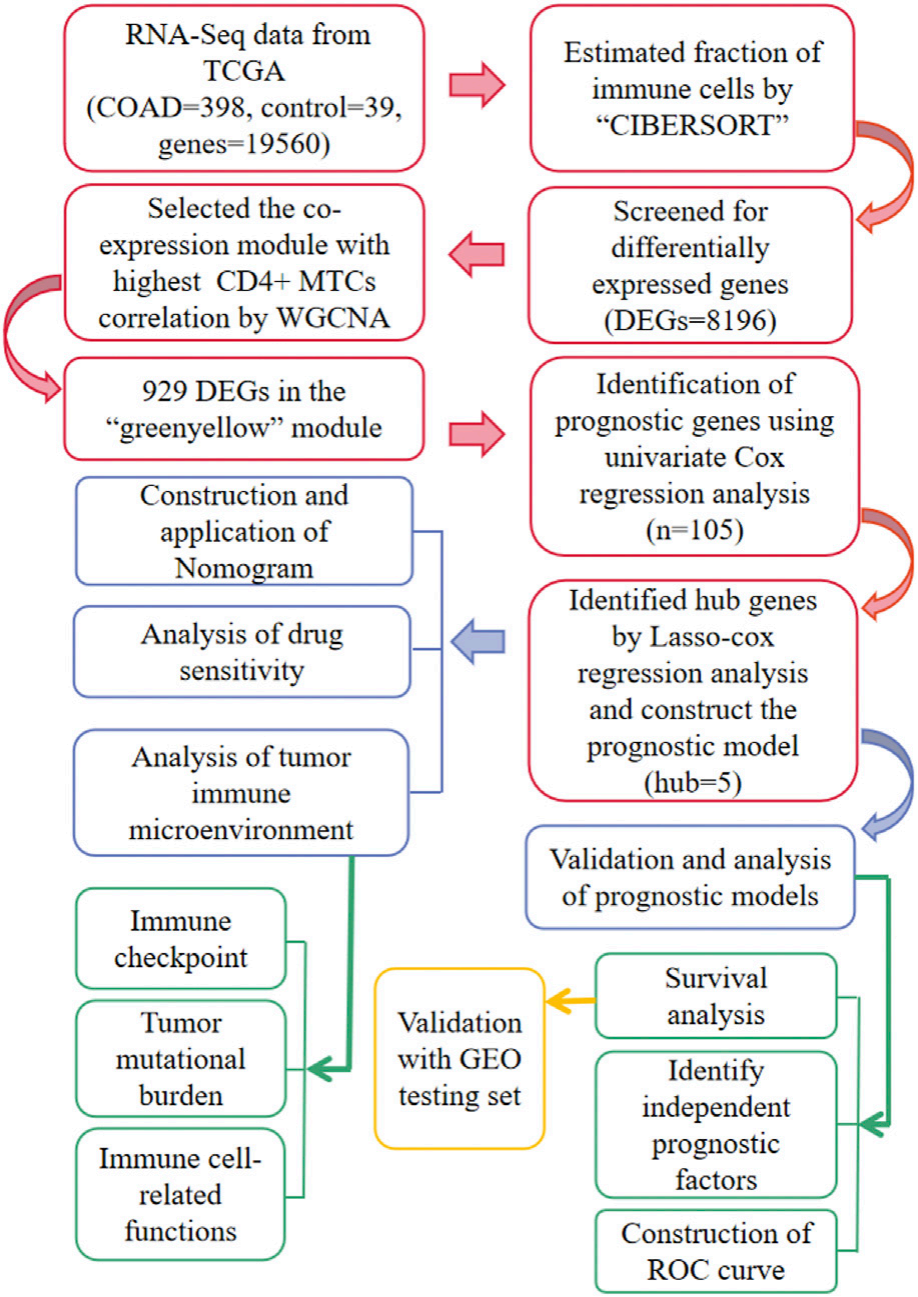
Workflow for the whole study.

The flowchart of the whole study is summarized in Figure 1.

**FIGURE 2:**
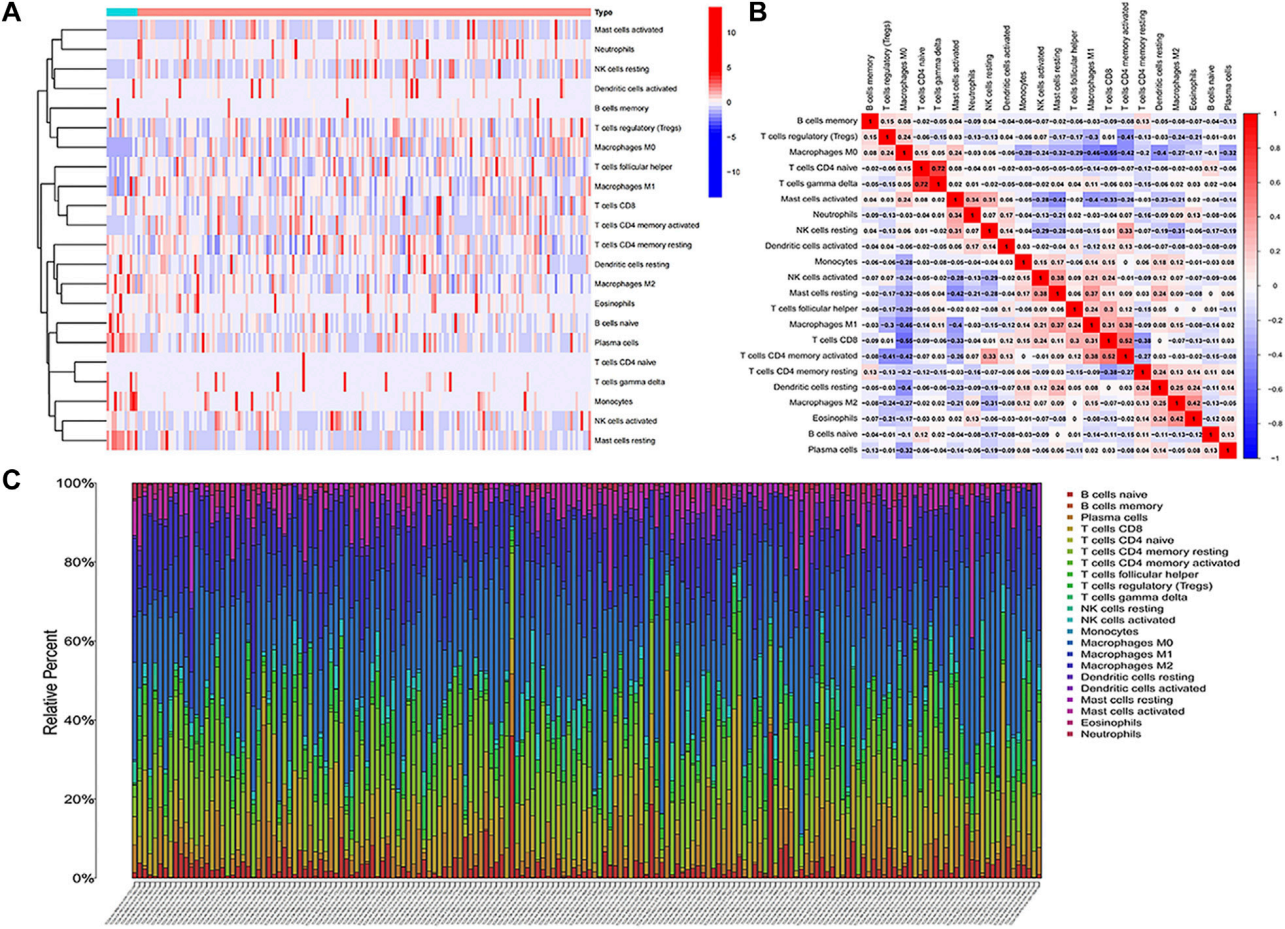
Analysis of immune cell infiltration. **(A)** Heatmap of the infiltration of 22 types of immune cells in COAD and normal samples. **(B)** Heatmap of the mutual infiltration relationship between immune cells in COAD samples. **(C)** Histogram of the infiltration proportion of immune cells in each COAD sample.

The RNA-Seq data of TCGA contained a total of 398 COAD and 39 normal samples, including 19,560 genes. After obtaining the expression matrix file, the proportion of 22 types of immune cell infiltration was determined by the “CIBERSORT” algorithm. Among the 22 types of immune cells, “Macrophages M0,” “T cells follicular helper,” “Macrophages M1,” and “T cells CD4 memory activated” were more actively expressed in COAD than in normal samples (Figure 2A). The heatmap shows the relationship between 22 immune cells in COAD (Figure 2B). The number in the small square is the Pearson product–moment correlation coefficient, which is used to measure the degree of linear correlation between variable X and variable Y. Its value is between −1 and 1, greater than 0 means positively correlated, and less than 0 means negatively correlated. 0 means that there is no linear correlation between the two variables. (For example, “B cells memory” and “Macrophages M0” are positively correlated, correlation coefficient = 0.08). Also, the bar plot shows the proportion of immune cell infiltration in each COAD. We can conclude that there was a higher proportion of T cells and macrophages (Figure 2C).

### Construction of the gene co-expression network

**FIGURE 3:**
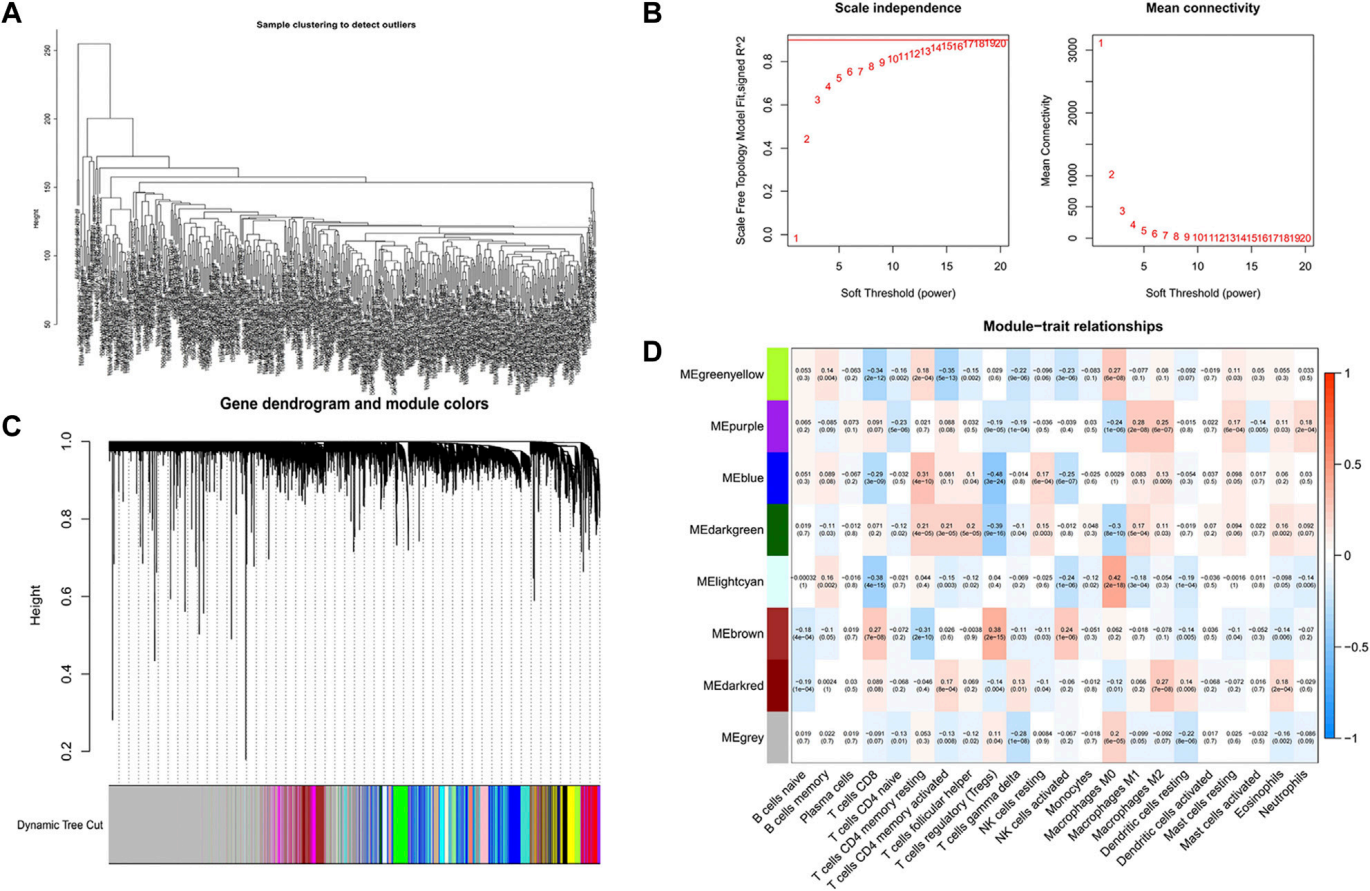
Screening of CD4^+^ MTC-related co-expression modules. **(A)** Sample clustering of WGCNA. **(B)** Scale-free fit index and average connectivity of the 1–20 soft threshold power (β) were analyzed. **(C)** Hierarchical clustering tree of genes based on the topological overlap. Different color branches of the cluster tree represent different modules. **(D)** Correlation between CD4^+^ MTCs and genes in each module.

We first analyzed all TCGA genes and extracted 8,196 DEGs between normal samples and COAD. Then, R software was used to eliminate the normal samples and genes with small fluctuation values in the data set and checked whether there is any deletion in the sample data and then removed the offending genes and samples from the data. Next, we detected outliers through sample clustering and eliminated them (Figure 3A). “sft$powerEstimate” function was used to determine the optimal soft-power threshold (*β* = 5), and the scale-free fit index of network topology was obtained by soft-thresholding power analysis (Figure 3B). Furthermore, we constructed a hierarchical clustering tree by using the dynamic shearing method and searched genes with similar expression data for modular clustering to generate a new hierarchical clustering tree (Figure 3C), and eight modules were generated. Finally, we draw the correlation plot between WGCNA modules and immune cells for further analysis (Figure 3D).

### Identification of hub genes and establishment of a prognostic model

**FIGURE 4:**
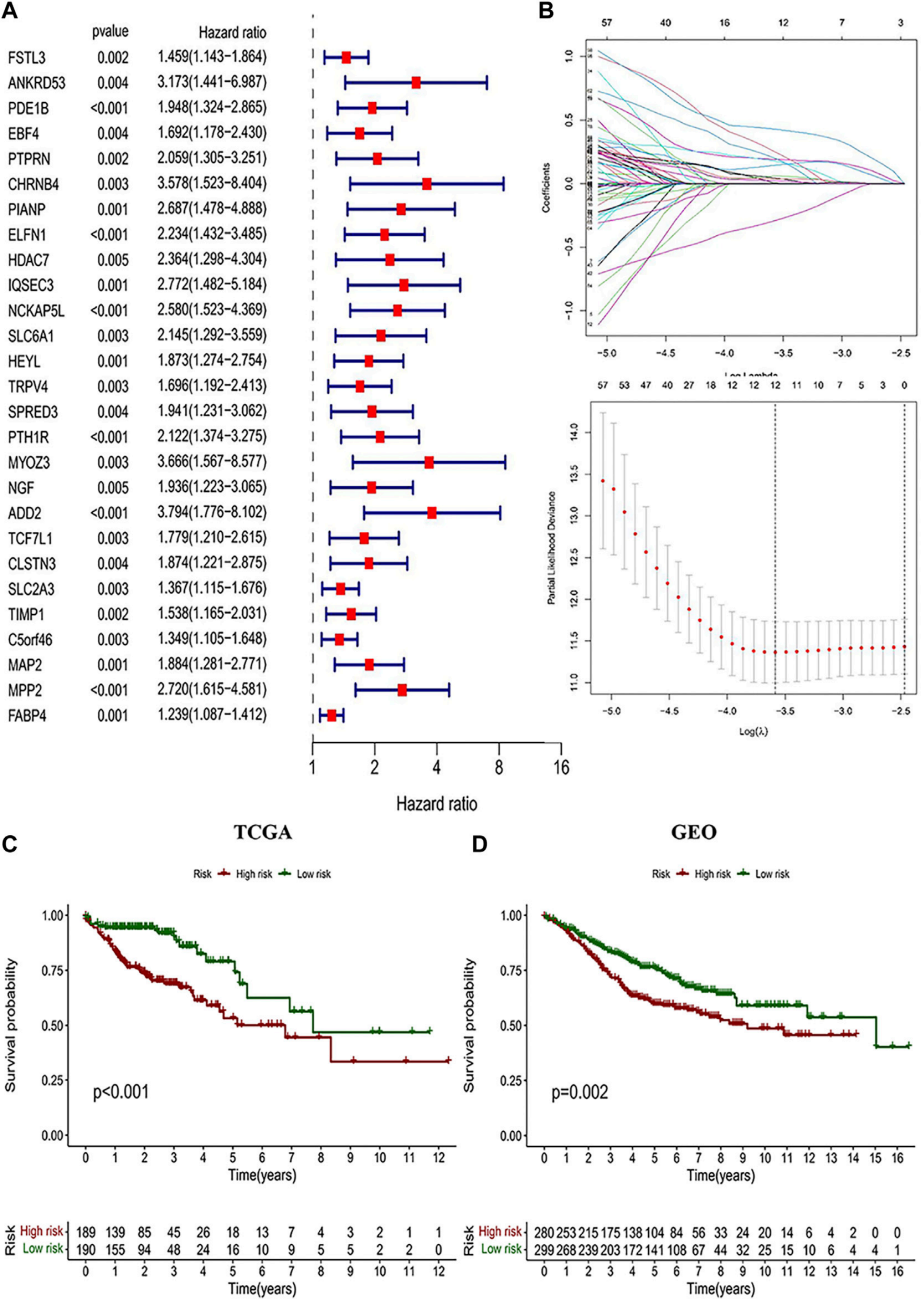
Identification of CD4^+^ MTC-related DEGs and construction of the prognosis model. **(A)** Univariate Cox regression analysis result of 103 prognosis-related genes in the study (27 genes with *p* < 0.005 were preferentially displayed). **(B)** Lasso regression and cross-validation showed that the number of genes corresponding to the point with the smallest error was 13. **(C)** COAD patients with low risk in the prognostic model predicted better OS outcomes. **(D)** GEO testing set was used for validation, and the results were consistent with those of the prognostic model.

The highest correlation with the CD4^+^ MTC-related gene was found in the “greenyellow” module (*R* ^2^ = −0.35, *p* < 0.0001), including 929 DEGs in total. According to the gene names in the “greenyellow” module, we searched in the TCGA and GEO sets respectively to extract expression levels of each gene. Also, gene expression files were merged with clinical data (Supplementary Table S2 and Supplementary Table S3) to prepare for further analysis. Univariate Cox analysis was performed on the “greenyellow” module genes and identified 105 CD4^+^ MTC-related DEGs associated with the OS of COAD (Supplementary Table S5), and we preferentially showed 27 DEGs with *p* < 0.005 in the forest plot (Figure 4A). We further identified 12 genes by LASSO regression analysis (Figure 4B). Then, a Cox model (Gill, 1982) was constructed based on these 12 genes, and finally, five hub genes (TGFB2, DTNA, S1PR5, F2RL2, and MPP2) and a model formula were obtained. The model formula was used to calculate the risk score of the training set: risk score = [TGFB2 × 0.320,583] + [F2RL2× (-0.612,387)] + [DTNA × 0.411,655] + [S1PR5 × 0.447,946] + [MPP2 × 0.718,418] (Table 1). According to the median value of the risk score, the samples of the training set were divided into high- and low-risk groups to predict the OS of COAD. Similarly, the samples of the testing set were also divided into two groups to verify the prediction accuracy of the prognostic model.

**TABLE 1:** Information and the corresponding coefficients of hub genes.

Gene symbol	Full name	Coefficient
F2RL2	Coagulation factor II (thrombin) receptor-like 2	−0.612,387
TGFB2	Transforming growth factor-β2	0.320,583
DTNA	Dystrobrevin-alpha	0.411,655
S1PR5	Sphingosine 1-phosphate receptor 5	0.447,946
MPP2	Membrane palmitoylated protein 2	0.718,418

### Predictive ability assessment of the prognostic model

**FIGURE 5:**
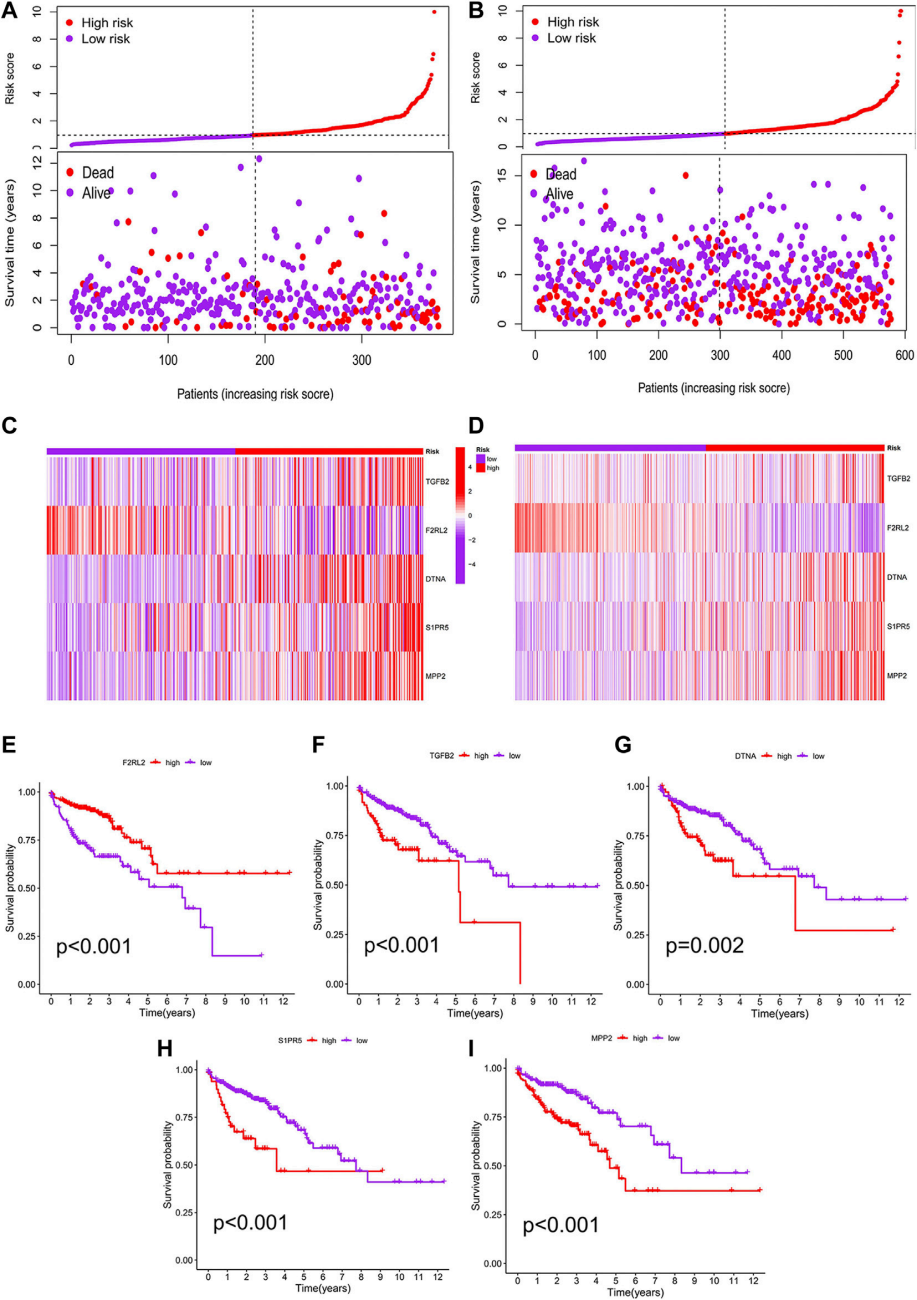
Correlation analysis between risk score and prognosis. **(A)** Patients in the prognostic model were divided into high- and low-risk groups according to the median value of risk score. With the increase in risk score, the number of deaths increased correspondingly, and the survival prognosis of patients with low risk was better. **(B)** Conclusions of the prognostic model were validated by the GEO testing set. **(C)** F2RL2 was confirmed to be a low-risk gene, while TGFB2, DTNA, S1PR5, and MPP2 were high-risk genes. **(D)** Conclusions of hub genes were validated by the GEO testing set. **(E–I)** K-M analysis results of five hub genes: F2RL2, TGFB2, DTNA, S1PR5, and MPP2.

We performed a K–M analysis between high- and low-risk groups on the prognostic model, and the result showed that patients with lower risk scores had a better outcome (*p* < 0.001, Figure 4C). This conclusion was also confirmed by the GEO testing set (*p* = 0.002, Figure 4D). As the risk score increased, the number of COAD patients who died increased accordingly, and patients at low-risk generally live longer than those at high risk (Figures 5A,B). In addition, in our prognostic model, F2RL2 was confirmed to be a low-risk gene, while TGFB2, DTNA, S1PR5, and MPP2 were high-risk genes (Figures 5C,D). K–M analysis proved that all five hub genes were closely related to OS of COAD, and patients with F2RL2 overexpression or SLC35G2, DTNA, S1PR5, and MPP2 low-expression had a better OS outcome (Figures 5E–I).

**FIGURE 6:**
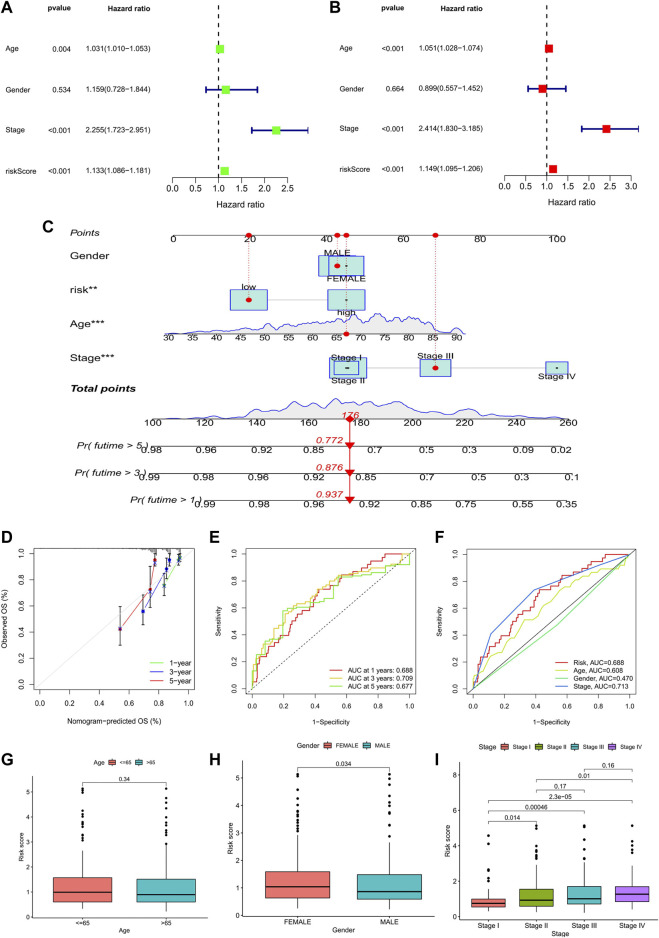
Analysis of prognostic factors and construction of nomogram. **(A)** Univariate analysis identified prognostic factors, and **(B)** multivariate analysis identified prognostic factors as independent predictors. **(C)** Nomogram used to predict the 1-, 3-, and 5-year survival rate in clinical medicine. (*p < 0.05, **p < 0.01,***p < 0.001). **(D)** Calibration curve to verify the accuracy of nomogram prediction. **(E)** ROC curves for OS prediction accuracy. **(F)** ROC curves for risk score and clinical features. **(G–I)** Analysis of clinical features between high- and low-risk groups in the prognostic model: age; gender; TNM stage.

Meanwhile, univariate and multivariate Cox analyses showed that age, stage, and risk score were independent prognostic factors associated with the OS of COAD (Figures 6A,B). Considering the application of the prognostic model in clinical practice, we developed nomograms to predict the OS at 1, 3, and 5 years based on the baseline characteristics and pathological parameters of COAD. Each COAD was individually matched with a nomogram, which fully embodied the individualization of clinical application. In the presented nomogram, the predicted 1-, 3-, and 5-year OS for this patient was 85.9%, 73.5%, and 54.7%, respectively (Figure 6C). Also, age, stage, and risk score were significantly associated with OS, which was consistent with the multivariate Cox analysis result. Subsequently, we use the calibration curve to verify the prediction accuracy of the nomogram. We can see that the prediction lines are very close to the diagonal dotted line, indicating that the nomogram has high accuracy (Figure 6D).

Furthermore, time-dependent-ROC analysis was performed to assess the predictive power of the risk score. Factors with an area under the curve (AUC) > 0.5 indicates that the prognostic model has a predictive value, and the greater the AUC, the higher the accuracy of prediction. Our analysis results showed that the prognostic model had good predictive value in predicting 1-, 3- and 5- year OS, and the prediction accuracy was 3- year (AUC: 0.720) >1- year (AUC: 0.718) >5- year (AUC: 0.692) (Figure 6E). Likewise, the predictive power of the prognostic model was superior to that of clinical predictors such as age and gender (Figure 6F).

Finally, we analyzed the clinical features between high- and low-risk groups in the prognostic model. The results showed that there were significant differences in gender and TNM stage between the two groups. The risk score of females was higher than that of males, and patients in stage IV had the highest risk score, while patients in stage I had the lowest risk score. However, it was worth noting that the risk scores between stage II and III (*p* = 0.17) and stage III and stage IV (*p* = 0.16) were not statistically significant (Figures 6G–I).

### Analysis of the tumor microenvironment and immune cell function

**FIGURE 7:**
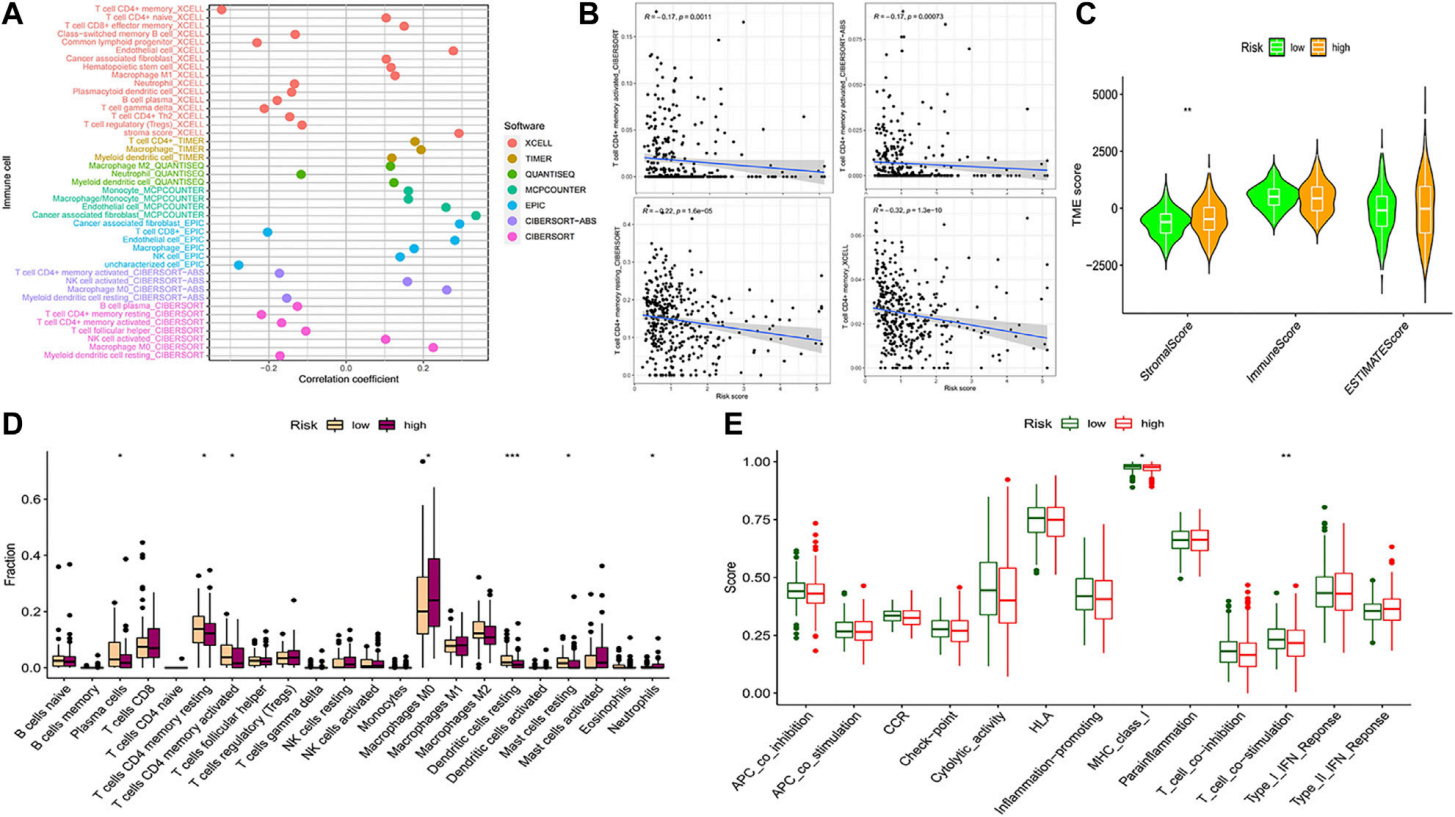
Analysis of immune cell infiltration and immune cell-related functions. **(A)** Bubble plot of immune cell infiltration related to the risk model was obtained by different software analyses. **(B)** Scatter plots of the correlation between the hub gene and CD4^+^ MTC infiltration. **(C)** Violin plot of tumor microenvironment score (**p < 0.001). **(D)** Differential analysis of immune cell infiltration in the prognostic model (*p < 0.05, **p < 0.01, and***p < 0.001). **(E)** Differential analysis of immune cell-related functions in the prognostic model (**p < 0.01).

We compared the prognostic model data with the pan-cancer immune cell infiltration data and analyzed the correlation between risk score, hub genes, and immune cell infiltration by R package “limma”. The bubble plot shows the results of the analysis performed by different software (Figure 7A). A correlation coefficient greater than zero is considered a positive correlation and that less than zero is a negative correlation. Similarly, the scatter plot suggested that CD4^+^ MTC-related DEGs were negatively correlated with immune scores (Figure 7B). In addition, we analyzed the association of the TME with risk scores. The COAD TME score was calculated by the ESTIMATE algorithm (estimation of stromal and immune cells in malignant tumor tissues using expression data). The violin plot reported significant differences in stromal cell scores between the high- and low-risk groups (*p* < 0.01), with higher scores in the high-risk group. But, there were no significant differences in immune cell score and ESTIMATE score between the two groups (Figure 7C).

Analysis of 22 types of immune cells showed that a total of seven types had significant differences in infiltration between the high- and low-risk groups (Figure 7D, *p* < 0.05). “Plasma cells”, “T cells CD4 memory resting”, “T cells CD4 memory activated”, “dendritic cells resting” and “mast cells resting” infiltration was upregulated in the low-risk group, while “macrophages M0” and “neutrophil” infiltration was upregulated in the high-risk group. In addition, analysis of immune cell-related functions showed that “T cell co−stimulation” and “MHC_class_I” functions were more active in the low-risk group (Figure 7E, *p* < 0.05).

### Immune checkpoint and immunotherapy

**FIGURE 8:**
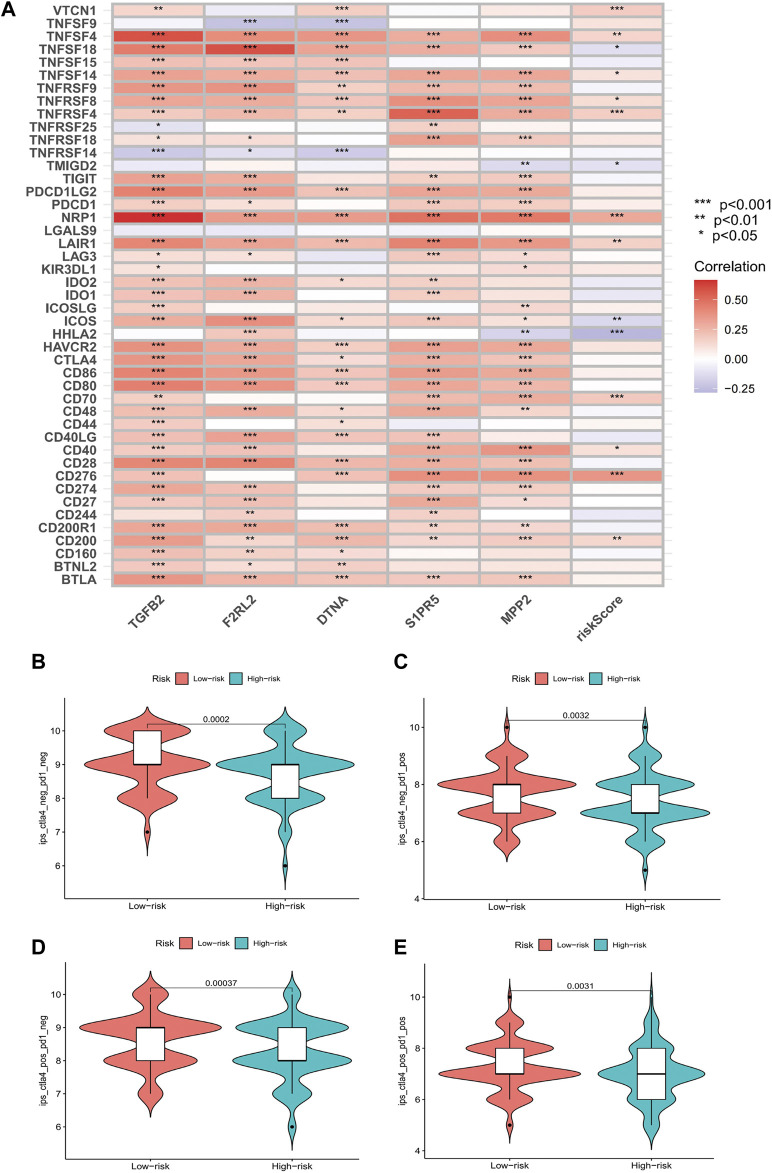
Results of immune checkpoints and immunotherapy efficacy analysis. **(A)** Heatmaps of immune checkpoints associated with hub genes and risk score (*p < 0.05, **p < 0.01, and***p < 0.001). **(B–E)** Violin plot of the association between immunotherapy effect and risk score: control group; anti-PD-1 group; anti-CTLA4 group; anti-PD-1/anti-CTLA4 group.

**FIGURE 9:**
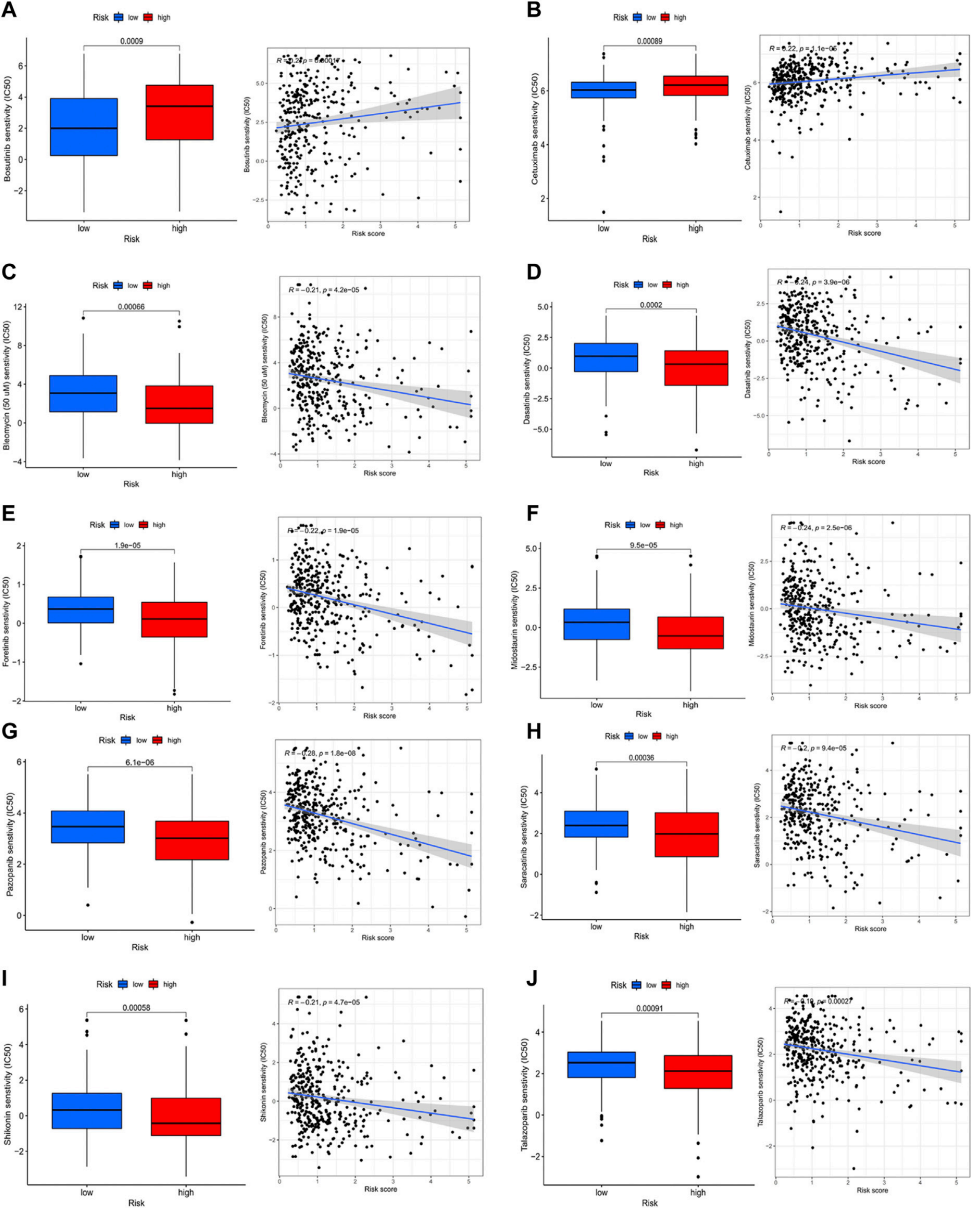
Analysis of drug sensitivity. **(A-j)** bosutinib; cetuximab; bleomycin (50 uM); dasatinib; foretinib; midostaurin; pazopanib; saracatinib; shikonin; talazoparib.

Comparing the hub genes and risk score with immune checkpoints to analyze their correlation, it is noteworthy to observe that immune checkpoints VTCN1, TNFSF4, TNFSF14, TNFRSF8, TNFRSF4, NRP1, LAIR1, CD40, CD70, CD276, and CD200 were positively correlated with risk scores, while TNFSF18, TMIGD2, ICOS, and HHLA2 were negatively correlated (Figure 8A). In the prediction of the therapeutic effect of immune checkpoint inhibitors (ICIs) in COAD, anti-CTLA-4 or anti-PD-1 or anti-CTLA-4 plus anti-PD-1 treatment was achieved better OS with a low-risk score (*p* < 0.005; Figures 8B–E). Meanwhile, we conducted a drug sensitivity analysis and concluded that bosutinib and cetuximab were more sensitive in COAD patients with low-risk scores (Figures 9A,B), while bleomycin (50 uM), dasatinib, foretinib, midostaurin, pazopanib, saracatinib, shikonin, and talazoparib were more sensitive in the high-risk score (Figures 9C–I). The specific mechanism of action and targeted pathway of these drugs can be searched in Genomics of Drug Sensitivity in Cancer (https://www.cancerrxgene.org/). Finally, we analyzed the association of TME with risk scores.

### Functional and pathway enrichment in the prognostic model

**FIGURE 10:**
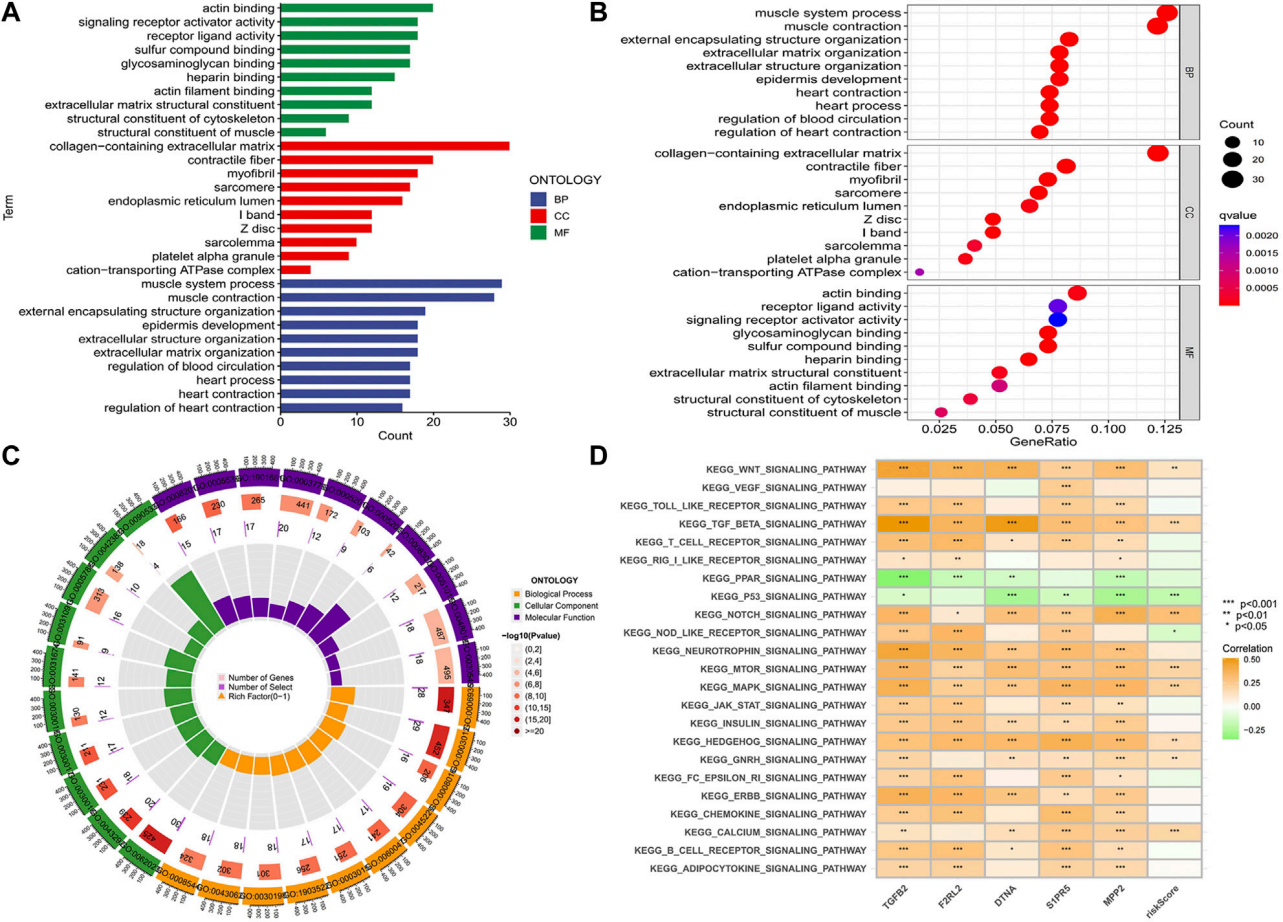
Functional and pathway enrichment analysis. **(A-C)** Histogram, bubble plot, and circle plot of GO enrichment analysis showed 30 functions that were significantly associated with differential risk genes and the number of genes enriched on each function. **(D)** Correlation of five hub genes and risk score with the KEGG pathway (*p < 0.05, **p < 0.01,***p < 0.001).

We conducted GO and KEGG enrichment according to the analysis results of DEGs between high- and low-risk groups. In the GO histogram (Figure 10A), we showed the number of risk-DEGs enriched in the three categories of GO (BP: Biological Process; CC: Cellular Component; MF: Molecular Function). In the GO bubble plot (Figure 10B), we showed the number and difference significance of the functional enrichment of risk-DEGs. In the circle plot (Figure 10C), the outermost circle represented the ID of GO, the second circle represented the number of genes enriched on each GO term, the third circle represented the number of DEGs enriched on each GO term, and the inner circle represented the proportion of genes. In the abovementioned plots, we showed 30 GO functions that were significantly associated with risk-DEGs and the number of genes enriched for each function. However, this was different from the results of GO enrichment analysis. In addition, we performed KEGG analyses of hub genes and risk scores to assess the association of these factors with the KEGG pathway and visualized the results with heat maps (Figure 10D).

### Association of tumor mutational burden with risk score

**FIGURE 11:**
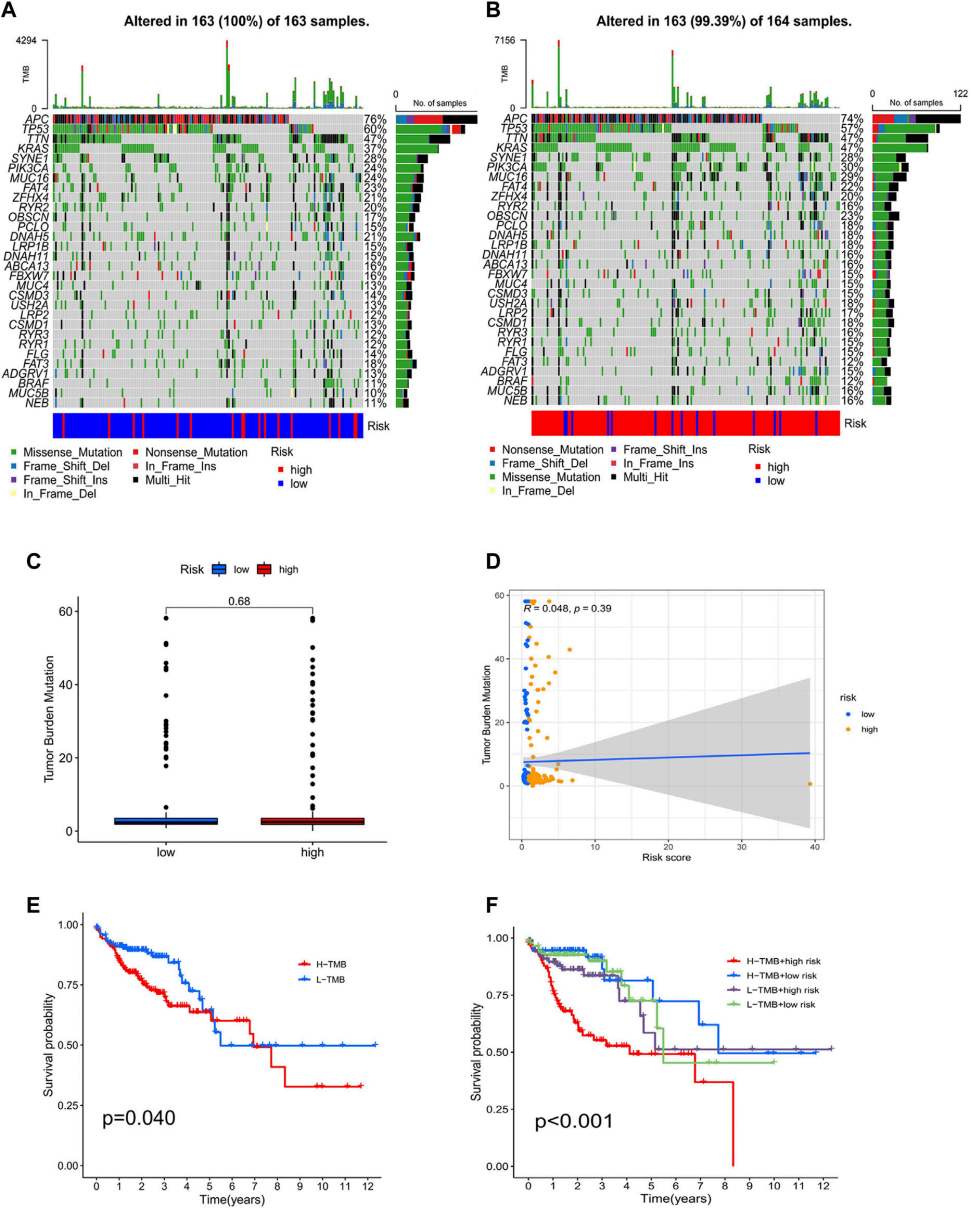
Analysis of tumor mutational burden. Mutations in the top 30 genes with the highest mutation frequency in low- **(A)** and high-risk groups **(B)**. Boxplots **(C)** and scatterplots **(D)** of the correlation between risk score and TMB. **(E)** K–M analysis of TMB. **(F)** K–M analysis of TMB combined with a risk score.

The TMB analysis was performed through R packages “BiocManager”, “ggpubr” and “maftools”. In the waterfall plot, we can observe that COAD in the low-risk group (100%) has higher gene mutation frequencies than that in the high-risk group (99.39%) and show the proportion of the top 30 genes with the highest mutation frequency (Figures 11A,B). However, the correlation analysis of TMB with risk score showed no significant difference between high- and low-risk groups (Figures 11C,D). Furthermore, K-M analysis was used to predict the OS of patients with TMB, and we observed that patients with low TMB have better OS (*p* = 0.04; Figure 11E). Also, we found that TMB combined with risk score had a significant difference in evaluating the prognosis of patients. Patients with high-TMB/low-risk had the best prognosis, followed by low-TMB/low-risk, low-TMB/high-risk, and high-TMB/high-risk (*p* < 0.001; Figure 11F).

## Discussion

Colorectal cancer is one of the most common types of malignancies, with the third highest morbidity and mortality in both males and females (Siegel et al., 2021). Although the overall morbidity and mortality have decreased year by year, the incidence has shown an upward trend in patients < 50 years of age (Cheng et al., 2011; Bailey et al., 2015; Siegel et al., 2017). Surgery, adjuvant chemotherapy, targeted therapy, and immunotherapy are the main options for treatment of colon cancer (Benson et al., 2018). With the younger age of colon cancer incidence population, the individualization and precision of treatment strategies are promising. Recent studies have pointed out that the TME plays a key role in cancer proliferation, invasion, and metastasis and helps predict the prognosis of the disease (Wang et al., 2021). CD4^+^ MTCs in the microenvironment can make a rapid and direct immune response to protect the host against the invasion of cancer cells (Hirahara et al., 2021). These findings suggest that it is a potential therapeutic target (Quail and Joyce, 2013).

In our study, we constructed a co-expression network based on gene expression in 398 TCGA-COAD samples. Through the “CIBERSORT” algorithm and WGCNA analysis, we preliminarily determined that the “greenyellow” module containing 929 DEGs was most significantly associated with CD4^+^ MTC infiltration. Subsequently, the optimal five hub genes (F2RL2, TGFB2, DNTA, S1PR5, and MPP2) were identified by univariate Cox-LASSO regression analysis, and model formulas were obtained. According to the model formula, TCGA-COAD patients were divided into high- and low-risk groups, and the prognostic risk model was constructed. In addition, K-M analysis was performed on the prognostic risk model to assess whether there was a significant difference in OS between the high- and low-risk groups. The result showed that patients in the low-risk group benefited more from OS and had a better prognosis than those in the high-risk group. More importantly, we validated this result with the GEO testing set, and K–M analysis also showed that patients in the low-risk group predicted better OS. This conclusion is consistent with the analysis results of our prognostic risk model, indicating that the TCGA prognostic risk model constructed by us has high accuracy in predicting the OS of COAD patients, which provides an important reference value for our clinical application.

F2RL2 is a G protein-coupled receptor that regulates protease-activated receptor-3 involved in inflammatory and immune responses (Zhou et al., 2019). It has been reported as a prognostic marker for oral squamous cell carcinoma (Huang SN et al., 2020), metastatic breast cancer (Liu et al., 2021), pancreatic cancer (Chen et al., 2022), and glioma (Lvu et al., 2020), and down-regulated F2RL2 expression has been detected in rectal cancer (Supiot et al., 2013). TGFB2 is a protein-coding gene of TGF-β2. It is considered to be the most critical factor in epithelial–mesenchymal transition and is involved in the key biological processes of growth, proliferation, migration, and invasion of malignant cells (Dave et al., 2011; Abraham et al., 2018; Yang et al., 2020), especially in suppressing antitumor immune response. Recent studies have shown that TGFB2 expression is upregulated in gastric cancer, non–small cell lung cancer, gallbladder cancer, colorectal carcinoma, and high-grade glioma, which is associated with poor prognosis (Bogdahn et al., 2011; Zhang et al., 2020; Liao et al., 2021; Song and Zhou, 2021). Therefore, targeted TGFB2 therapy for cancer patients may be a promising strategy. Currently, several therapies that specifically inhibit TGFB2, such as antisense phosphorothioate oligodeoxynucleotide trabedersen (AP12009), have entered clinical development in patients with advanced cancers (Jaschinski et al., 2011). DTNA is a scaffold protein that maintains the structural integrity of the heart and skeletal muscle (Cao et al., 2017) and has been proven to predict the survival prognosis of bladder cancer (Zhang et al., 2021), hepatocellular carcinoma (Huang SN et al., 2020), gastric adenocarcinoma (Qin et al., 2019) and esophageal cancer (Fu et al., 2021). Liu et al. (Liu et al., 2017) also found that DTNA had a reference value in early colon cancer screening. S1PR5 is a G protein-coupled receptor, belonging to one of the five subtypes of S1PRs, which is distributed in many tissues and cells of the human body, especially in immune cells (Takabe and Spiegel, 2014; Patmanathan et al., 2017). Also, d Zhou et al. showed that up-regulation of S1PR5 could activate the NF-κB/IDO1 signaling pathway and promote the progression of colon cancer (Zhou H et al., 2021). MPP2 is a scaffold protein belonging to the membrane-associated guanylate kinase-P55 (MAGUK) subfamily. MPP2 is closely associated with cell adhesion and is critical for the formation of multiprotein complexes involved in cell–cell communication. MPP2 is known for its junctional function in epithelial cells (Baumgartner et al., 2009; Rademacher et al., 2016). At present, the role and mechanism of MPP2 in cancer are rarely reported. In the only two *in vitro* studies on MPP2, Li et al. (2021) confirmed that MPP2 can be up-regulated through miR-34a demethylation, promoted liver cancer cell apoptosis, and reduced proliferation, migration, and invasion. Similarly, Maschietto et al.reported that the MPP2 gene was downregulated in relapse Wilms tumors (Maschietto et al., 2011). In our prognostic risk model, overexpression of MPP2 was associated with a higher risk score and worse OS. Although this conclusion is different from the abovementioned results of liver cancer or Wilms tumors, there is no relevant report on the role of MPP2 in the occurrence and development of colon cancer. Therefore, the relationship between MPP2 and colon cancer and its specific mechanism remains to be explored.

Our findings showed that F2RL2 was downregulated in high-risk patients and considered to be a protective gene, while TGFB2, DTNA, S1PR5, and MPP2 were up-regulated in high-risk patients, suggesting that they were associated with poor prognosis. In our risk model, age, stage, and risk score were independent prognostic factors for COAD. The older the age, the higher the risk score, the higher the cancer stage, and the worse the prognosis. According to the gene expression level in the selected panel, we drew a nomogram to evaluate the survival time of patients. Each sample can get the corresponding nomogram through software, which is almost cost-free, portable, and intuitive in clinical applications.

Current treatments for colon cancer include surgery, chemotherapy, and targeted therapy. However, existing clinical studies have reported that EGFR inhibitor treatment is not conducive to long-term survival and disease remission (Cunningham et al., 2004; Lièvre et al., 2006; Van Emburgh et al., 2014). Therefore, the exploration of immunotherapy is highly expected. ICI therapy has shown promising results in melanoma and lung cancer (Lichtenstern et al., 2020). In 2019, ICI drugs were approved for breast cancer (Schmid et al., 2020). However, there are few reports of other cancers benefiting from ICI therapy. Nowadays, there is no large-scale transcriptome data for colon cancer immunotherapy, so we use RNA-seq in the TCGA database to calculate Immunophenoscore and then verify the effectiveness of ICI treatment. Our analysis found that anti-CTLA-4 or anti-PD-1 or anti-CTLA-4/anti-PD-1 therapy was more effective in low-risk patients. In addition, we screened for immune checkpoints associated with risk scores. These results provide an idea for future immunotherapy studies and drug selection.

Although our CD4^+^ MTC infiltration prognosis model has achieved some important results, it must be admitted that there are still shortcomings. First, this is a retrospective analysis using the public database, and we still lack validation of prospective studies. In addition, our study considered the influence of immune infiltration and gene mutation on the progression of colon cancer, but there are many other epigenetic modifications in the pathology of the disease. An analysis combining these factors may be of greater reference value. Furthermore, in future studies, the potential mechanisms of action associated with hub genes in our prognostic risk model need to be further validated *in vivo* and *in vitro*. It is worth mentioning that when using the GEO dataset to validate the TCGA prognostic risk model, the selection of different datasets may bring some deviation to our validation results. For example, the sample size of the GEO dataset is insufficient or some GEO datasets may study a specific race or different disease stages (AJCC stage or TNM stage) may be used among different GEO datasets. Therefore, in order to make the validation results more reliable, we need to consider the clinical characteristics and sample size of the samples comprehensively when selecting the GEO dataset.

## Conclusion

In summary, we constructed a risk assessment model of five CD4^+^ MTC-related gene markers for COAD and drew the nomogram of the hub gene and clinical independent risk factors to assess immunotherapy efficacy, disease prognosis, and survival time of the patient. These results provide a reference for target selection and individualized immunotherapy of COAD.

## Data Availability

The original contributions presented in the study are included in the article/Supplementary Material; further inquiries can be directed to the corresponding author.

## Abbreviations

COAD, colon adenocarcinoma; OS, overall survival; TME, tumor microenvironment; CD4^+^ MTCs, CD4^+^ memory T cells; WGCNA, weighted gene co-expression network analysis; LASSO, the least absolute shrinkage, and selection operator; DEGs, differentially expressed genes; TCGA, the Cancer Genome Atlas database; GEO, Gene Expression Omnibus; TCIA, the Cancer Immunome Database; TMB, tumor mutational burden; CTLA-4, cytotoxic T-lymphocyte-associated protein 4; PD-1, programmed cell death protein 1; FDR, false discovery rate, K-M, Kaplan–Meier; ROC, receiver operating characteristic; IC_50_, half-maximal inhibitory concentration; GO, Gene Ontology; KEGG, Kyoto Encyclopedia of Genes and Genomes; AUC, area under the curve; ICIs, immune checkpoint inhibitors.
