# Supplementary material for: Identification of hub genes related to CD4+ memory T cell infiltration with gene co-expression network predicts prognosis and immunotherapy effect in colon adenocarcinoma
Source: Front Genet. 2022 Aug 29;13:915282. doi: 10.3389/fgene.2022.915282 (PMC9465611; doi:10.3389/fgene.2022.915282)
Supplement: Supplementary file 3 [file Table1.DOCX]

**Supplementary Table 1. TCGA database retrieval strategy**

| Data type | Retrieval strategy |
| --- | --- |
| Transcriptome data | cases. Disease type in [“adenomas and adenocarcinomas”] and cases. Primary site in [“colon”] and cases Project.program.name in [“TCGA”] and cases Project id in [“TCGA-COAD”] and files. Access in [“open”] and files. Data category in [“transcriptome profiling”] and files. Data type in [“Gene Expression Quantification”] and files. Experimental strategy in [“RNA-Seq”] |
| Clinical data | cases. Disease type in [“adenomas and adenocarcinomas”] and cases. Primary site in [“colon”] and cases Project.program.name in [“TCGA”] and cases Project id in [“TCGA-COAD”] and files. Access in [“open”] and files. Data category in [“clinical”] and files. Data type in [“Clinical Supplement”] and files. Data format in [“bcr xml”] |
| Gene mutation data | cases. Disease type in [“adenomas and adenocarcinomas”] and cases. Primary site in [“colon”] and cases Project.program.name in [“TCGA”] and cases Project id in [“TCGA-COAD”] and files. Access in [“open”] and files. Data category in [“simple nucleotide variation”] and files. Data type in [“Masked Somatic Mutation”] and files. Data format in [“maf”] |

**Note:** Age at diagnosis, race, gender and survival state of all the above data were not restricted.
