# Supplementary material for: Identification of hub genes related to CD4+ memory T cell infiltration with gene co-expression network predicts prognosis and immunotherapy effect in colon adenocarcinoma
Source: Front Genet. 2022 Aug 29;13:915282. doi: 10.3389/fgene.2022.915282 (PMC9465611; doi:10.3389/fgene.2022.915282)
Supplement: Supplementary file 4 [file Table7.DOCX]

**Supplementary Table 7. Script for prognostic model construction**

| #install.packages("survival")  library(survival)  pFilter=0.05  setwd("C:\\Users\\Desktop\\ File")  rt=read.table("SupplementaryTable2", header=T, sep="\t", check.names=F, row.names=1)  rt$futime=rt$futime/365  # Univariate Cox regression analysis  outTab=data.frame()  sigGenes=c("futime","fustat")  for(gene in colnames(rt[,3:ncol(rt)])){  cox=coxph(Surv(futime, fustat) ~ rt[,gene], data = rt)  coxSummary = summary(cox)  coxP=coxSummary$coefficients[,"Pr(>\|z\|)"]    if(coxP<pFilter){  sigGenes=c(sigGenes,gene)  outTab=rbind(outTab,  cbind(gene=gene,  HR=coxSummary$conf.int[,"exp(coef)"],  HR.95L=coxSummary$conf.int[,"lower .95"],  HR.95H=coxSummary$conf.int[,"upper .95"],  pvalue=coxP) )  }  }  write.table(outTab,file="uniCox.txt",sep="\t",row.names=F,quote=F)  surSigExp=rt[,sigGenes]  surSigExp=cbind(id=row.names(surSigExp), surSigExp)  write.table(surSigExp,file="uniSigExp.txt",sep="\t",row.names=F,quote=F) |
| --- |
| install.packages("glmnet")  install.packages("survival")  set.seed(123456)  library("glmnet")  library("survival")  coxSigFile="uniSigExp.txt"  setwd("C:\\Users\\Desktop\\ File ")  rt=read.table(coxSigFile, header=T, sep="\t", check.names=F, row.names=1)  rt$futime[rt$futime<=0]=0.003  # LASSO regression analysis  x=as.matrix(rt[,c(3:ncol(rt))])  y=data.matrix(Surv(rt$futime, rt$fustat))  fit=glmnet(x, y, family="cox", maxit=1000)  #Figure of LASSO regression analysis  pdf("lasso.lambda.pdf")  plot(fit, xvar="lambda", label=TRUE)  dev.off()  #Figure of cross validation  cvfit=cv.glmnet(x, y, family="cox", maxit=1000)  pdf("lasso.cvfit.pdf")  plot(cvfit)  abline(v=log(c(cvfit$lambda.min,cvfit$lambda.1se)), lty="dashed")  dev.off()  coef=coef(fit, s=cvfit$lambda.min)  index=which(coef != 0)  actCoef=coef[index]  lassoGene=row.names(coef)[index]  lassoSigExp=rt[,c("futime", "fustat", lassoGene)]  lassoSigExpOut=cbind(id=row.names(lassoSigExp), lassoSigExp)  write.table(lassoSigExpOut,file="lasso.SigExp.txt",sep="\t",row.names=F,quote=F)  # Cox model Constructed  multiCox=coxph(Surv(futime, fustat) ~ ., data = lassoSigExp)  multiCox=step(multiCox,direction = "both")  multiCoxSum=summary(multiCox)  # Model formula  outMultiTab=data.frame()  outMultiTab=cbind(  coef=multiCoxSum$coefficients[,"coef"],  HR=multiCoxSum$conf.int[,"exp(coef)"],  HR.95L=multiCoxSum$conf.int[,"lower .95"],  HR.95H=multiCoxSum$conf.int[,"upper .95"],  pvalue=multiCoxSum$coefficients[,"Pr(>\|z\|)"])  outMultiTab=cbind(id=row.names(outMultiTab), outMultiTab)  outMultiTab=outMultiTab[,1:2]  write.table(outMultiTab, file="multiCox.txt", sep="\t", row.names=F, quote=F)  trainScore=predict(multiCox, type="risk", newdata=rt)  coxGene=rownames(multiCoxSum$coefficients)  coxGene=gsub("`","",coxGene)  outCol=c("futime","fustat",coxGene)  risk=as.vector(ifelse(trainScore>median(trainScore),"high","low"))  outTab=cbind(rt[,outCol],riskScore=as.vector(trainScore),risk)  write.table(cbind(id=rownames(outTab),outTab),file="risk.TCGA.txt",sep="\t",quote=F,row.names=F) |
